# Identification of spin effects in the anomalous Righi–Leduc effect in ferromagnetic metals

**DOI:** 10.1038/s41598-020-68669-w

**Published:** 2020-07-16

**Authors:** Da-Kun Zhou, Qing-Lian Xu, Xiao-Qin Yu, Zhen-Gang Zhu, Gang Su

**Affiliations:** 1 School of Electronic, Electrical and Communication Engineering, University of Chinese Academy of Sciences, Beijing, 100049 China; 2 Theoretical Condensed Matter Physics and Computational Materials Physics Laboratory, College of Physical Sciences, University of Chinese Academy of Sciences, Beijing, 100049 China; 3 School of Physics and Electronics, Hunan University, Changsha, 410082 China; 4 CAS Center for Excellence in Topological Quantum Computation, University of Chinese Academy of Sciences, Beijing, 100190 China; 5 Kavli Institute of Theoretical Sciences, University of Chinese Academy of Sciences, Beijing, 100049 China

**Keywords:** Physics, Spintronics

## Abstract

The emerging of spin caloritronics leads to a series of new spin-thermal related effects, such as spin Seebeck effect (SSE), spin Nernst effect (SNE) and their corresponding inverse effects. Anomalous Righi–Leduc effect (ARLE) describes that a transverse temperature gradient can be induced by a longitudinal heat flow in ferromagnets. The driving force and the response of the ARLE are all involved with heat. It is curious if spin effects mediate the heat transport and provide extra influence. In this work, we investigate the ARLE and the interplay between the heat current, charge current, and spin current via linear response theory. We identified that spin effects do have clear roles in heat transport, which can be confirmed by phase shifts of voltage output varying with the direction of magnetization. Our formulas fit the experimental data very well. Moreover, we discuss more configuration of magnetization which is expected to be tested in the future. It should be emphasized that the present formalism including spin effects is out of the theory based on magnon transport, which may be conspicuous in the devices within the spin diffusion length.

## Introduction

Spin caloritronics^1–3^, which is an extension and combination of spintronics and conventional thermoelectrics, has recently emerged as a research area. Spin caloritronics study the non-equilibrium transport phenomena involving the interplay between spin, charge, entropy and energy in mostly magnetic structures and devices. Spin caloritronic phenomena can be roughly classified into three categories^4^. The first class is the collective effects which are generated by the collective dynamics of the magnetic order parameter that couple to a single spin, such as spin Seebeck effect. The second class is the independent electron effects which are thermoelectric generalization of collinear magnetoelectronics and effects, such as spin-dependent Peltier effect. The third class is the relativistic effects which are thermoelectric generation of relativistic corrections, such as spin Hall effect (SHE).

The SHE^5,6^ describes generation of dissipationless spin current in a transverse direction due to an applied electric field in the longitudinal direction in materials with strong spin-orbit coupling in absence of magnetic field. Recently, the inverse effect to SHE was proposed, i.e. inverse spin Hall effect (ISHE)^7,8^, in which the injected longitudinal spin current can be converted into a measured transverse electric field or a voltage drop. In 2008, Uchida et al.^9^ proposed a new longitudinal effect, i.e. spin Seebeck effect (SSE) which leads to the emerging of spin-caloritronics. The SSE^10–12^ has been suggested to be a way for spin-current-generation (or spin accumulation) as a consequence of a temperature gradient due to the spin splitting of density of states. Recently, spin Nernst effect (SNE) was proposed and studied^13–19^, which is a Hall-like effect in which temperature gradient is applied as a driving force instead of the electric field.

In view of the developments of spin caloritronics, some conventional effects are revisited^20^, such as Righi–Leduc effect. The conventional Righi–Leduc effect (thermal Hall effect)^21,22^ describes a Hall-like transverse heat-current-generation in response to a longitudinal driving temperature gradient in presence of perpendicular external magnetic field $\mathbf {B}$. In ferromagnets, a transverse temperature gradient can be generated by applying a longitudinal temperature gradient even at zero $\mathbf {B}$, which is called anomalous Righi–Leduc effect (ARLE)^23,24^. Based on magnon transport (particularly for ferromagnetic insulators), Wegrowe et al.^25^ found that as the direction of magnetization changed (through the external magnetic field), the transverse temperature gradient also changed periodically, that is, there was an angular dependence between the transverse temperature gradient and the direction of magnetization.

In principle, carriers carry not only heat but also spin in ferromagnetic materials. Thus the spin effects may play roles in the heat transport. However, the study of the spin effects on the heat transport is sparse. The reason may be that the spin effects are covered up by heat degree of freedom in analysis of the data in experiments. From the perspective of complete basic knowledge of heat transport, it is desired to explore if there are spin effects on the heat transport and how large they are. In this work, we start from a full description of the heat transport including the charge, spin and heat flows for ferromagnetic metals so that our theory can be compared with some experiments. Our calculation reveals that spin plays multi-functional roles in the heat transport and can be identified from experimental data. We show that the transverse temperature gradient is strongly dependent on the orientation of magnetization. Then, in order to fit the experimental data in Ref.^23^, we used thermocouple effect to transform the temperature gradient at both ends of the electrode into the electric potential. Finally, we use the formula of total voltage to fit with the experimental data and found that it fits well. The spin effects may be more prominent in smaller devices within the spin diffusion length.

The paper is composed of three parts. In the first part, we derived the general formula. In the second part, We fit the experimental data in terms of the general formulas, and then give some analysis. In the last part, we give some discussions and conclusions.

## Formalism

The ARLE describes an thermal phenomenon in which heat current can be generated in the *y* direction as a response to the applied temperature gradient in the *x* direction when the magnetization of ferromagnet is along the *z* direction. The magnitude of the effect can be characterized by the anomalous Righi–Leduc coefficient (ARLC) $\lambda _{\text {RL}}$, which is determined by the ratio of the generated transverse heat current density $J_{y}^{q}$ to the applied transverse temperature gradient $\nabla _{x}T$^26^

1 $$\begin{aligned} \lambda _\text {RL}=\frac{J_{y}^{q}}{\nabla _{x}T}, \end{aligned}$$

with the condition

2 $$\begin{aligned} J_{x}=J_{y}=\nabla _{y}T=0, \end{aligned}$$

where $J_{x}$ and $J_{y}$ are electric current densities.

In the linear response theory, each component of particle (heat) current density is a linear function of thermodynamic driving forces. In our study, we extend the theory by taking into account the spin degree of freedom and spin current densities as extra response of thermodynamic driving forces. Thus, in the ferromagnets, the spin-dependent linear response equations are expressed as^14^

3 $$\begin{aligned} \left( \begin{array}{c} \frac{\mathbf {J}^{s}}{h/2\pi } \\ \mathbf {J}^{n}\\ \mathbf {J}^{q} \end{array}\right) =\left( \begin{array}{ccc} \mathbf {L}^{ss} &{} \mathbf {L}^{sn} &{} \mathbf {L}^{sq}\\ \mathbf {L}^{ns} &{} \mathbf {L}^{nn} &{} \mathbf {L}^{nq}\\ \mathbf {L}^{qs} &{} \mathbf {L}^{qn} &{} \mathbf {L}^{qq} \end{array}\right) \left( \begin{array}{c} -\varvec{\nabla }\mu ^{s}\\ -\varvec{\nabla }\mu ^{n}\\ -\frac{\varvec{\nabla }T}{T} \end{array}\right) , \end{aligned}$$

where spin potential gradient $-\varvec{\nabla }\mu ^{s}$, electrochemical potential gradient $\varvec{\nabla }\mu ^{n}$ and $\frac{\varvec{\nabla }T}{T}$ are the corresponding thermodynamic driving forces to spin current density $\mathbf {J}^{s}$, particle current density $\mathbf {J}^{n}$ and heat current density $\mathbf {J}^{q}$, respectively, and *T* is the temperature. In Eq. (3), we have $\mathbf {L}^{ij}=\mathbf {L}^{ji} (i\ne j)$ satisfying the Onsager relation^14,27,28^. And the coefficient $\mathbf {L}$ entailed by the symmetry in isotropic materials, i.e., $L^{ij}_{xx}=L^{ij}_{yy}$ and $L^{ij}_{xy}=-L^{ij}_{yx}$.

In open circuit case, after reaching steady state, there is no electrical current flowing $\mathbf {J}=e \mathbf {J}^{n}=0$. Thus, the spin-dependent linear response equations (Eq. (3)) can be written as follows

4 $$\begin{aligned} \frac{\mathbf {J}^{s}}{h/2\pi }= & {} \mathbf {A}^{ss}\left( -\varvec{\nabla }\mu ^{s}\right) +\mathbf {A}^{sq}\left( -\frac{\nabla T}{T}\right) , \end{aligned}$$

5 $$\begin{aligned} \mathbf {J}^{q}= & {} \mathbf {A}^{qs}\left( -\varvec{\nabla }\mu ^{s}\right) +\mathbf {A}^{qq}\left( -\frac{\nabla T}{T}\right) , \end{aligned}$$

with

6 $$\begin{aligned} \mathbf {A}^{ss}= & {} \mathbf {L}^{ss}-\mathbf {L}^{sn}\left( \mathbf {L}^{nn}\right) ^{-1}\mathbf {L}^{ns},\nonumber \\ \mathbf {A}^{sq}= & {} \mathbf {L}^{sq}-\mathbf {L}^{sn}\left( \mathbf {L}^{nn}\right) ^{-1}\mathbf {L}^{nq},\nonumber \\ \mathbf {A}^{qq}= & {} \mathbf {L}^{qq}-\mathbf {L}^{qn}\left( \mathbf {L}^{nn}\right) ^{-1}\mathbf {L}^{nq},\nonumber \\ \mathbf {A}^{qs}= & {} \mathbf {L}^{qs}-\mathbf {L}^{qn}\left( \mathbf {L}^{nn}\right) ^{-1}\mathbf {L}^{ns}, \end{aligned}$$

where $\left(h/2\pi\right)\mathbf{A}^{ss}=-\left(\mathbf{J}^{s}/\nabla\mu^{s}\right)_{\nabla T=0}$ is spin conductance tensor. $\left( h/2\pi \right) \mathbf {A}^{sq}/T=-(\mathbf {J}^{s}/\nabla T)_{\nabla\mu^{s}=0}$ describes the spin current generated by temperature gradient and its diagonal (off-diagonal) terms characterize the spin Seebeck (Nernst) effect. $\mathbf {A}^{qs}=-(\mathbf {J}^{q}/\varvec{\nabla }\mu ^{s})_{\nabla T=0}$, illustrating inverse effects towards $\mathbf {A}^{qs}$, describes the heat current generated by spin chemical potential.

According to Eqs. (4) and (5), heat current density can be determined by spin current and temperature gradient as

7 $$\begin{aligned} \mathbf {J}^{q}=\bar{\varvec{\Pi }}^{s}\left( \frac{\mathbf {J}^{s}}{\left( h/2\pi \right) }\right) +\bar{\varvec{\lambda }}\left( -\frac{\varvec{\nabla } T}{T}\right) , \end{aligned}$$

where coefficient $\bar{\varvec{\Pi }}^{s}=\left(h/2\pi\right)(\mathbf {J}^{q}/\mathbf {J}^{s})_{\nabla T=0}=\mathbf {A} ^{qs}\left( \mathbf {A}^{ss}\right) ^{-1}$ is spin Peltier tensor, which shows the interplay between spin current and heat current. The coefficient $\bar{\varvec{\lambda }}=-(\mathbf {J}^{q}/{\nabla } T)_{\mathbf {J}^{s}=0}=\left[ \mathbf {A} ^{qq}-\mathbf {A}^{qs}\left( \mathbf {A}^{ss}\right) ^{-1}\mathbf {A}^{sq}\right]$ is generalized thermal conductivity tensor^20^. And the generalized thermal conductivity tensor includes two terms. The first term is coming from the diagonal components of $\mathbf {A}^{qq}$ which describes the generated heat current by a temperature gradient. Therefore it is actually the coefficient of conventional Fourier law. The second term, i.e. $\mathbf {A}^{qs}\left( \mathbf {A}^{ss}\right) ^{-1}\mathbf {A}^{sq}$, describes an additional effect stemming from a generated spin current by a temperature gradient and mutually driving heat current. Therefore, this term is a novel contribution firstly derived in Ref.^20^.

**Figure 1:**
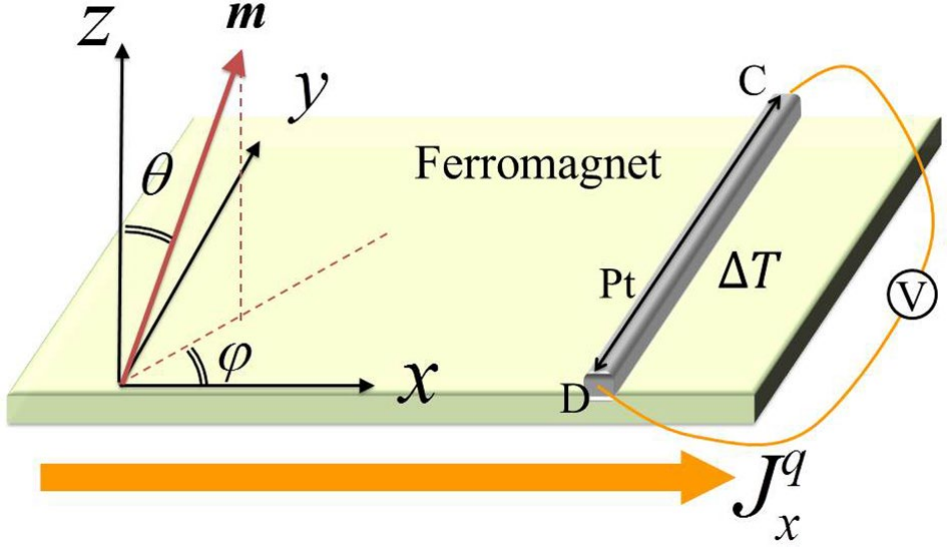
Schematic view of the magnetization $\mathbf {m}\left( \theta ,\varphi \right)$ in the ferromagnet.

In isotropic materials, $\bar{\varvec{\Pi }}^{s}$ and $\bar{\varvec{\lambda }}$ follow the certain symmetry. If the ferromagnet is placed in *x*–*y* plane and the magnetization direction is along the *z* direction, the matrix form of coefficient $\bar{\varvec{\Pi } }^{s}$ is:

8 $$\begin{aligned} \bar{\varvec{\Pi } }^{s}=\left( \begin{array}{ccc} \Pi ^{s} &{} \left( pt\right) ^{s} &{} 0\\ -\left( pt\right) ^{s} &{} \Pi ^{s} &{} 0\\ 0 &{} 0 &{} \Pi _{z}^{s} \end{array}\right) , \end{aligned}$$

where $\Pi ^{s}$ is the spin Peltier coefficient and $\left( pt\right) ^{s}$ is a transverse effect coefficient which quantifies the heat current generated by perpendicular spin current in absence of temperature gradient. According to the magnetic anisotropy, we have $\Pi ^{s} \ne \Pi _{z}^{s}$. The matrix form of coefficient $\bar{\varvec{\lambda }}$ is:

9 $$\begin{aligned} \bar{\varvec{\lambda }}=\left( \begin{array}{ccc} \kappa &{} \lambda _\text {RL} &{} 0\\ -\lambda _\text {RL} &{} \kappa &{} 0\\ 0 &{} 0 &{} \kappa _{z} \end{array}\right) , \end{aligned}$$

where $\kappa$ is the thermal conductivity and $\lambda _\text {RL}$ is the anomalous Righi–Leduc coefficient in the spin system. Similarly, due to the magnetic anisotropy $\kappa \ne \kappa _{z}$. Since the tensor $\bar{\varvec{\lambda }}$ is composed of two parts, the matrix element of $\bar{\varvec{\lambda }}$ is also composed of two parts. The first term is actually the coefficient of conventional Fourier law. The second term is coming from spin-thermal effect.

In experiments, the direction of the magnetization is usually varied by applying an external magnetic field. The angular dependence of the measurements not only gives us more information about the physical systems, but also may exclude some accompanying effects to those that we want to study. Therefore it is instructive to investigate the specific angular dependence. The spatial orientation of magnetization $\mathbf {m}\left( \theta ,\varphi \right)$ is characterized by azimuth angle $\varphi$ and polar angle $\theta$ (see Fig. 1). Thus, the heat current in Eq. (7) can be rewritten as ^25^

10 $$\begin{aligned} \mathbf {J}^{q}= & {} \left[ \Pi ^{s}{\hat{\mathbf {I}}}+\Delta \Pi ^{s}{\hat{\mathbf {P}}}_{m}-\left( pt\right) ^{s}{\hat{\mathbf {Q}}}_{m}\right] \left( \frac{\mathbf {J}^{s}}{\left( h/2\pi \right) }\right) \nonumber \\&+\left[ \kappa {\hat{\mathbf {I}}}+\Delta \kappa {\hat{\mathbf {P}}}_{m}-\lambda _\text {RL}{\hat{\mathbf {Q}}}_{m}\right] {\left( -\frac{\varvec{\nabla } T}{T}\right) }, \end{aligned}$$

where $\Delta \Pi ^{s}=\Pi _{z}^{s}-\Pi ^{s}$ and $\Delta \kappa =\kappa _{z}-\kappa$. ${\hat{\mathbf {I}}}$ is the identity matrix and ${\hat{\mathbf {P}}}_{m}\equiv \mathbf {m}\otimes \mathbf {m}^{t}$ is defined as the direct product of the magnetization vector, where $\mathbf {m}^{t}$ is the transpose of $\mathbf {m}$. ${\hat{\mathbf {Q}}}_{m}$ is the antisymmetric representation of $\mathbf {m}$, corresponding to an operation ${\hat{\mathbf {Q}}}_{m}$: $\mathbf {X}\mapsto \mathbf {m}\times \mathbf {X}$ (The explicit expressions of matrices ${\hat{\mathbf {P}}}_{m}$ and ${\hat{\mathbf {Q}}}_{m}$ are given in Appendix.).

According to Eq. (10), temperature gradient can be rewritten as function of heat current and spin current, i.e.,

11 $$\begin{aligned} \frac{\varvec{\nabla } T}{T} & = \left[ r{\hat{\mathbf {I}}}+\left( \triangle r\right) {\hat{\mathbf {P}}}_{m}+r_\text {RL}{\hat{\mathbf {Q}}}_{m}\right] \left( -\mathbf {J}^{q}\right) \nonumber \\&\quad+\left[ \Pi ^{s}r+\left( pt\right) ^{s}r_\text {RL}\right] {\hat{\mathbf {I}}}\left( \frac{\mathbf {J}^{s}}{\left( h/2\pi \right) }\right) \nonumber \\&\quad+\left[ \Pi _{z}^{s}r_{z}-\Pi ^{s}r-\left( pt\right) ^{s}r_\text {RL}\right] {\hat{\mathbf {P}}}_{m}\left( \frac{\mathbf {J}^{s}}{\left( h/2\pi \right) }\right) \nonumber \\&\quad-\left[ \left( pt\right) ^{s}r-\Pi ^{s}r_\text {RL}\right] {\hat{\mathbf {Q}}}_{m}\left( \frac{\mathbf {J}^{s}}{\left( h/2\pi \right) }\right) \nonumber \\ & = \mathbf {C}_{1}\left( -\mathbf {J}^{q}\right) +\mathbf {C}_{2}\left( \frac{\mathbf {J}^{s}}{\left( h/2\pi \right) }\right) , \end{aligned}$$

where $\mathbf {C}_{1}$ and $\mathbf {C}_{2}$ are two parameter matrices, $r={\kappa }/{\left[ \left( \kappa \right) ^{2}+\left( \lambda _\text {RL}\right) ^{2}\right] }$ is thermal resistivity in the *x*, *y* directions, $r_{z}={1}/{\kappa _{z}}$ is thermal resistivity in the *z* direction and $r_\text {RL}={\lambda _\text {RL}}/{\left[ \left( \kappa \right) ^{2}+\left( \lambda _\text {RL}\right) ^{2}\right] }$ is Righi–Leduc resistivity. $\Delta r=\left( {1}/{\kappa _{z}}\right) -r$ represents the anisotropy of the thermal resistance. It can be found that the contribution of heat current to temperature gradient is determined by the coefficients ($\kappa ,\lambda _\text {RL}$) in the thermal resistance tensor $\bar{\varvec{\lambda }}$. The contribution from the spin current to the temperature gradient is the result of the interaction between effects described by the spin Peltier tensor $\bar{\varvec{\Pi }}^{s}$ and those characterized by $\bar{\varvec{\lambda }}$. We first consider the case in which the magnetization vector is fixed at *z* direction. Therefore we get

$$\begin{aligned} \mathbf {C}_{2}= \left( \begin{array}{lll} \Pi ^{s}r+\left( pt\right) ^{s}r_\text {RL} &{} \left( pt\right) ^{s}r-\Pi ^{s}r_\text {RL} &{} 0\\ -\left( pt\right) ^{s}r+\Pi ^{s}r_\text {RL} &{}\Pi ^{s}r+\left( pt\right) ^{s}r_\text {RL} &{} 0\\ 0 &{} 0 &{} \prod _{z}^{s}r_\text {z} \end{array}\right) , \end{aligned}$$

where the first two diagonal terms are the same, i.e. $\Pi ^{s}r+\left( pt\right) ^{s}r_\text {RL}$. The reason is that we study isotropic materials. For anisotropic sample, the two terms are generally different to each other. Two distinct mechanisms in deriving the diagonal term can be identified: a longitudinal–longitudinal joint effect (LLJE) ($\Pi ^{s}r$), and a transverse–transverse joint effect (TTJE) ($\left( pt\right) ^{s}r_\text {RL}$). In the LLJE, a longitudinal heat current is firstly generated by spin current through the spin Peltier effect and then is converted into the temperature gradient through the thermal resistivity. The TTJE describes such a combined processes: a spin current in the *x* direction generates a *y* direction heat current which induces another heat current in the *x* direction again by the anomalous Righi–Leduc effect. Therefore it appears in the diagonal position in the matrix of $\mathbf {C}_{2}$. It’s remarkable that the generated temperature gradient is still parallel to spin current as that in the LLJE.

The off-diagonal terms in $\mathbf {C}_{2}$ are $\pm \left( pt\right) ^{s}r\mp \Pi ^{s}r_\text {RL}$ which involves two different longitudinal-transverse joint effects. One longitudinal-transverse joint effect is characterized by $\left( pt\right) ^{s}r$ and describes that a transverse heat current can be generated when applying a spin current in longitudinal direction and then will be converted into temperature gradient via thermal resistance. The other longitudinal–transverse effect is expressed by $\Pi ^{s}r_\text {RL}$ which illustrates that heat current is firstly generated by spin current via spin Peltier effect and then is converted into the transverse temperature gradient through anomalous Righi–Leduc effect. Unlike the LLJE and TTJE, the generated temperature gradient and applied spin current are perpendicular to each other in both longitudinal–transverse joint effect. Thus, the off-diagonal terms describe transverse effects in which transverse temperature gradient is generated by the spin current. In summary, the contribution of the spin current to the generated temperature gradient is indirect and is the combination of various spin thermoelectric effects.

Since the magnetization rotates in real space, the magnetization $\mathbf {m}$ can be characterized as $\mathbf {m}=m_{x}\mathbf {x}+m_{y}\mathbf {y}+m_{z}\mathbf {z}$ and $m_{x}=\sin \theta \cos \varphi$, $m_{y}=\sin \theta \sin \varphi$ and $m_{z}=\cos \theta$. When the heat current flows along *x* direction (Fig. 1), after reaching steady state, the heat flow in the y and z directions is zero. So, the generated temperature gradient in the *y* direction reads

12 $$\begin{aligned} \frac{\nabla _{y}T}{T}= & {} \left[ \left( \Pi _{z}^{s}r_{z}-\Pi ^{s}r-\left( pt\right) ^{s}r_\text {RL}\right) \frac{J_{x}^{s}}{\left( h/2\pi \right) }-\triangle rJ_{x}^{q}\right] \sin ^{2}\theta \cos \varphi \sin \varphi \nonumber \\&+\left( \Pi _{z}^{s}r_{z}-\Pi ^{s}r-\left( pt\right) ^{s}r_\text {RL}\right) \frac{J_{y}^{s}}{\left( h/2\pi \right) }\left( \sin \theta \sin \varphi \right) ^{2}\nonumber \\&-\left[ \left( \left( pt\right) ^{s}r-\Pi ^{s}r_\text {RL}\right) \frac{J_{x}^{s}}{\left( h/2\pi \right) }+r_\text {RL}J_{x}^{q}\right] \cos \theta \nonumber \\&+\left( \Pi ^{s}r+\left( pt\right) ^{s}r_\text {RL}\right) \frac{J_{y}^{s}}{\left( h/2\pi \right) }. \end{aligned}$$

Equation (12) is the specific expression of transverse temperature gradient obtained by using linear response theory based on electron transport. It can be seen that there are three angle-dependent terms and the last term without explicit angular dependence. Compared to Eq. (16) in Ref.^25^, there are two more terms which are the term of $(\sin \theta \sin \varphi )^{2}$ and the last term. In fact, the corresponding coefficients of these two terms contain $J_{y}^{s}$, and the corresponding coefficients of the other two angle terms are $J_{x}^{s}$ and $J_{x}^{q}$. Usually, $J_{x}^{s}$ and $J_{x}^{q}$ may be larger than $J_{y}^{s}$, so $(\sin \theta \sin \varphi )^{2}$-terms and the last term can be ignored in most materials. In this way, the angular dependence based on electron transport is consistent with that based on magnon transport. This does not mean that these extra terms stemming from the spin effects are not important. In principle, the spin effects do exist theoretically. And their realistic roles may be important in some materials in which spin effects are not ignorable. In “Fitting and analysis” section, we use our formula to fit the experimental data in Ref.^23^, which give better fits than the formula derived without spin effect. In brief, we confirm the existence of the spin caloritronic effects. In some materials, the relatively large spin Nernst effect may allow these extra terms to be retained. In Ref.^29^, Satya et al. have observed a remarkably large anomalous Nernst effect (ANE) in the ferromagnetic topological Heusler compound $\hbox {Co}_2$MnGa, which is $\sim 7$ times larger than any value reported for conventional ferromagnets to date in the literature. And they revealed that this high value of the ANE arises from a large Berry curvature. Similar to ANE, with a large Berry curvature, we can also get a large spin Nernst effect ^15^. It may be argued that the spin caloritronic effects discussed in this work may be more important in such a case. It is worth noting that Eq. (12) corresponds to the case in which the heat flow in the y-direction is zero. For a comparison, we show below the situation of non-zero y-direction heat flow. Thus the Eq. (12) should be modified (in terms of Eq. (11)) as

13 $$\begin{aligned} \begin{aligned} \frac{\nabla _{y} T}{T}&=\left[ \left( \Pi _{z}^{s} r_{z}-\Pi ^{s} r-(p t)^{s} r_{\mathrm {RL}}\right) \frac{J_{x}^{s}}{\left( h/2\pi \right) r}-\Delta r J_{x}^{q}\right] \sin ^{2} \theta \cos \varphi \sin \varphi \\&\quad +\left[ \left( \Pi _{z}^{s} r_{z}-\Pi ^{s} r-(p t)^{s} r_{\mathrm {RL}}\right) \frac{J_{y}^{s}}{\left( h/2\pi \right) }-\Delta r J_{y}^{q}\right] (\sin \theta \sin \varphi )^{2} \\&\quad -\left[ \left( (p t)^{s} r-\Pi ^{s} r_{\mathrm {RL}}\right) \frac{J_{x}^{s}}{\left( h/2\pi \right) }+r_{\mathrm {RL}} J_{x}^{q}\right] \cos \theta \\&\quad +\left[ \left( \Pi ^{s} r+(p t)^{s} r_{\mathrm {RL}}\right) \frac{J_{y}^{s}}{\left( h/2\pi \right) } -r J_{y}^{q}\right] . \end{aligned} \end{aligned}$$

Comparing the above formula to Eq. (12), it is seen that there is an additional $J_{y}^{q}$ term in the $\sin ^{2}\theta \sin ^{2} \varphi$-term and the constant term. However, introducing the extra $J_{y}^{q}$ heat current does not bring additional angular dependence. To identify the existence of spin-thermal effects experimentally, a non-zero $J_{y}^{q}$ should be avoided. Fortunately, this is just the case studied in the experiment^23^ where open circuit condition is applied along y direction. Therefore, we can identify the role of spin-thermal effects by identifying the extra angular dependence.

## Fitting and analysis

We have deduced the general formula of the transverse temperature gradient in “Formalism” section, and in this section we will use our formula to fit the experimental data in experiment^23^. Because the temperature difference in the *y* direction is obtained by measuring the voltage at both ends of the electrode by thermocouple, i.e. the C and D positions in Fig. 1, we need to express the temperature difference in the *y* direction into voltage drop at C and D points. The temperature difference along the *y* direction in the ferromagnet causes the temperature difference at both ends of the electrode by heat conduction. The linear response on the electrode is as follows: $\mathbf {J}=-\frac{\varvec{\sigma }}{q} \varvec{\nabla } \mu ^{e}-\varvec{\sigma } \mathbf {S} \varvec{\nabla } T$, where $\mathbf {J}$ is the charge current, $\mu ^{e}$ is the electrochemical potential, $\varvec{\sigma }$ is the conductance tensor and $\varvec{S}$ is the Seebeck tensor. In open circuit situation, i.e. $\mathbf {J}=0$, the electrochemical potential gradient is expressed in terms of the temperature gradient as $-\frac{1}{q} \varvec{\nabla } \mu ^{e}=\mathbf {S} \varvec{\nabla } T$. And the integration of electrochemical potential gradients is the potential difference, i.e. $\Delta V_{y}=-\int _{C}^{D} \frac{\partial \mu ^{e}}{q \partial y} d y$^25^. The potential difference in the *y* direction can be expressed as (detailed derivation is in Appendix):

14 $$\begin{aligned} \Delta V_{y}=\Delta S \Delta _{y} T+S_{N} \Delta _{x} T , \end{aligned}$$

where $\Delta _{x} T=\int _{C}^{D} \frac{\partial T}{\partial x} d y$, $\Delta _{y} T=\int _{C}^{D} \frac{\partial T}{\partial y} d y$. $\Delta S$ is the Seebeck coefficient difference between electrodes and the wires connected to the voltmeter in Fig. 1, $S_{N}$ is the Nernst coefficient for electrode. Usually the Nernst effect is much smaller than the Seebeck effect, thus only the first item is considered, i.e. $\Delta V_{y}=\Delta S \Delta _{y} T$. So we get

15 $$\begin{aligned} \Delta V_{y} & = A \Delta S \left\{ \left[ \left( \Pi _{z}^{s} r_{z}-\Pi ^{s} r-(p t)^{s} r_{R L}\right) \frac{J_{x}^{s}}{\left( h/2\pi \right) }-\Delta r J_{x}^{q}\right] \sin ^{2}\theta \cos \varphi \sin \varphi \right. \nonumber \\&\quad+\left[ \left( \Pi _{z}^{s} r_{z}-\Pi ^{s} r-(p t)^{s} r_{R L}\right) \frac{J_{y}^{s}}{\left( h/2\pi \right) }\right] \sin ^{2}\theta \sin ^{2}\varphi \nonumber \\&\quad+\left[ -\left( (p t)^{s} r-\Pi ^{s} r_{R L}\right) \frac{J_{x}^{s}}{\left( h/2\pi \right) }-r_{R L} J_{x}^{q}\right] \cos \theta \nonumber \\&\quad+ \left. \left[ \left( \Pi ^{s} r+(p t)^{s} r_{R L}\right) \frac{J_{y}^{s}}{\left( h/2\pi \right) }\right] \right\} , \end{aligned}$$

where $$A=\int _{C}^{D} T(y) d y$$.

Next, we will fit the experimental data for the magnetization in *x*–*y* plane configuration and in *y*–*z* plane configuration respectively. In addition, we will also analyze *x*–*z* configuration, which was not measured in the experiment in Ref.^23^. However, before fitting, it is important to notice that the angles $\theta$ and $\varphi$ in Eq. (15) describe the magnetization and not of the magnetic field ($\theta _{H}$ and $\varphi _{H}$). The relation between external magnetic field (i.e., $\theta _{H}$, $\varphi _{H}$) and magnetization (i.e., $\theta$, $\varphi$) is given by the minimization of the ferromagnetic free energy. The concrete expression of ferromagnetic free energy will be given when discussing the *y*–*z* configuration.

**Figure 2:**
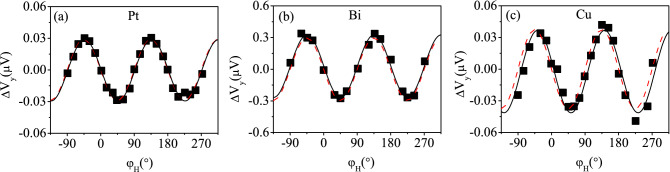
Relationship between transverse voltages $\Delta V_{y}$ and the direction of external magnetic field $\varphi _H$ in *x*–*y* configuration. The black spot is the experimental data, the dashed line is the fitting curve in Ref.^23^, and the solid lines are the fitting curve obtained by Eq. (16). The electrodes are (**a**) Pt, (**b**) Bi and (**c**) Cu respectively.

### Magnetization in x–y plane

When magnetization in *x*–*y* plane, we have $\theta _{H}=\theta$ = 90 °C. The direction of magnetization $\varphi =\varphi _{H}$ can be obtained by calculating the minimum ferromagnetic free energy. Now, the voltage in the *y* direction is

16 $$\begin{aligned} \Delta V_{y}= & {} \left\{ \left[ \left( \Pi _{z}^{s} r_{z}-\Pi ^{s} r-(p t)^{s} r_{R L}\right) \frac{J_{x}^{s}}{\left( h/2\pi \right) r} -\left( \triangle r\right) J_{x}^{q}\right] \right. \nonumber \\&\times \cos \varphi \sin \varphi \nonumber \\&+\left[ \left( \Pi _{z}^{s} r_{z}-\Pi ^{s} r-(p t)^{s} r_{R L}\right) \frac{J_{y}^{s}}{\left( h/2\pi \right) }\right] \sin ^{2}\varphi \nonumber \\&+\left. \left[ \left( \Pi ^{s} r+(p t)^{s} r_{R L}\right) \frac{J_{y}^{s}}{\left( h/2\pi \right) }\right] \right\} A \Delta S. \end{aligned}$$

**Figure 3:**
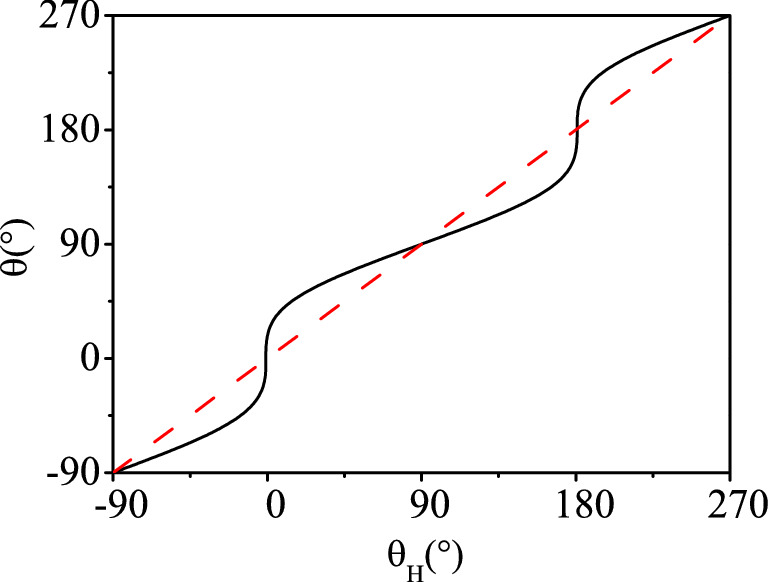
This figure shows the relationship between $\theta$ and $\theta _{H}$ derived by performing the minimum of free energy. The solid line represents the numerically calculated relationship between $\theta$ and $\theta _{H}$. As a reference, we show the relation of $\theta =\theta _{H}$ by a dashed line.

**Figure 4:**
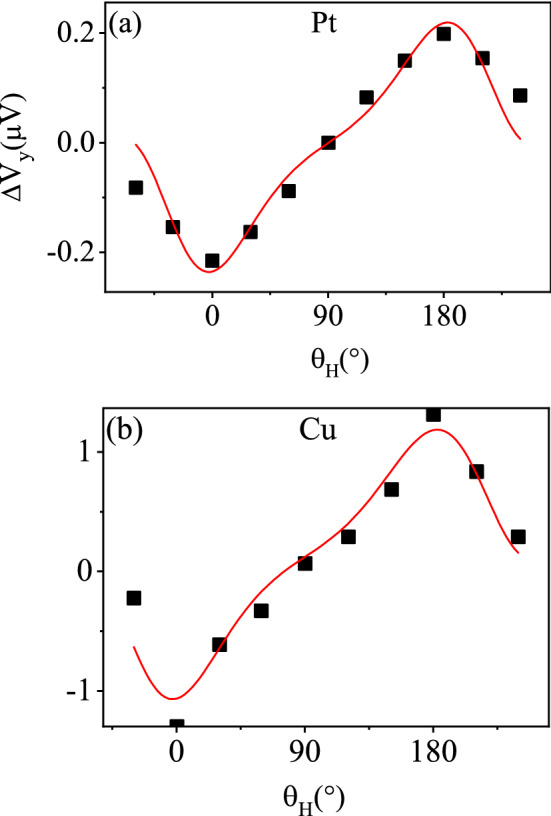
Relationship between transverse voltages $\Delta V_{y}$ and the direction of external magnetic field $\theta _H$ in *y*–*z* configuration. The black spot is the experimental data, the solid lines are the fitting curve. The electrodes are (**a**) Pt and (**b**) Cu respectively.

When a heat current is flowing in *x* direction, a spin current will be generated in the same direction due to the SSE, aligning the polarization of spin current along magnetization (in *x*–*y* plane). A voltage drop will be developed at the ends of the electrode (C and D point) due to the spin current injection into the electrode and the corresponding ISHE in it with large spin orbit coupling (such as Pt, Bi). However, it seems that no signal of the $2\pi$ periodicity of ISHE was observed in the experiment^23^. Therefore, we directly use Eq. (16) to fit the experimental data, without considering the influence of ISHE.

In Fig. 2, the theoretical fitting curves to experiments for Pt, Bi and Cu electrodes are given respectively. The black dots indicate the experimental data, the dashed lines are the fitting curves given in Ref.^23^, and the solid lines are the fitting curves in the present work obtained from Eq. (16). It is necessary to explain the formula in Ref.^23^, which is $\Delta V_{y} \approx J_{x}^{Q} \Delta S \Delta r \cos \varphi \sin \varphi$. This equation is based on magnons transport. For a comparison, our formula Eq. (16) based on electron transport. It can be seen that the solid lines have certain phase shift with respect to the dashed lines. The phase shift for each curve is caused by the extra angle term and the last term (see Eq. (16)). For Cu, the phase shift is $\tan (\phi )=0.264$. For Bi, the phase shift is $\tan (\phi )=-\ 0.084$. Although the phase shifts are small for Pt and Bi electrodes, it can be clearly seen that the phase shift is quite significant when Cu is used as the electrode. If we use $\hbox {Co}_2\hbox {MnGa}$ mentioned in “Formalism” section as the experimental material replacing *NiFe*, and then use Eq. (16) and formula in Ref. ^23^ to fit the experimental data respectively, then we will hopefully see a larger phase shift. In other words, it is not proper to ignore the two extra terms stemming from the spin effects.

### Magnetization in *y*–*z* plane

**Figure 5:**
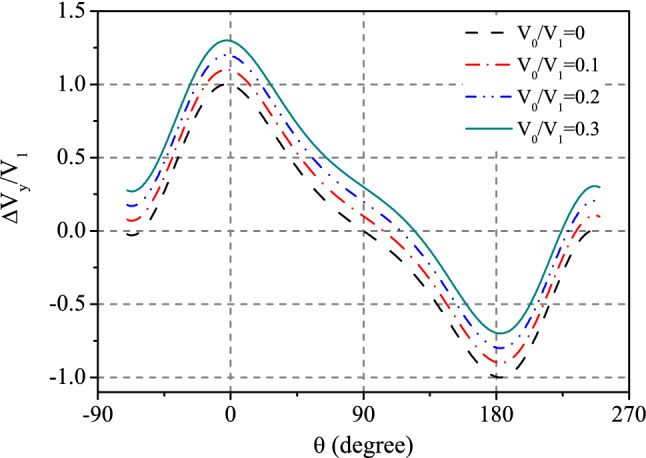
Relationship between transverse voltages $\Delta V_{y}$ and the direction of external magnetic field $\theta _H$ in *x*–*z* configuration. The formula we use is $\frac{\Delta V_{y}}{V_{1}}=\cos \theta +V_{0}/V_{1}$, which is a shorthand for Eq. (19).

In this section, we now discuss the configuration of magnetization in *y*–*z* plane, i.e. $\varphi _{H}=\varphi =90^{\circ }$. The voltage in the *y* direction is then

17 $$\begin{aligned} \Delta V_{y}&=A \Delta S\left\{ \left[ \left( \Pi _{z}^{s} r_{z}-\Pi ^{s} r-(pt)^{s} r_{RL}\right) \frac{J_{y}^{s}}{\left( h/2\pi \right) }\right] \sin ^{2}\theta \right. \nonumber \\&\quad +\left[ -\left( (pt)^{s} r-\Pi ^{s} r_{RL}\right) \frac{J_{x}^{s}}{\left( h/2\pi \right) }-r_{RL} J_{x}^{q}\right] \cos \theta \nonumber \\&\quad +\left. \left[ \left( \Pi ^{s} r+(pt)^{s} r_{RL}\right) \frac{J_{y}^{s}}{\left( h/2\pi \right) }\right] \right\} , \end{aligned}$$

where $\theta$ is the magnetization direction. Since the experimental data measure the voltages $\Delta V_{\mathrm {y}}$ as a function of $\theta _{H}$ (the angle of external magnetic field), we need to relate $\theta$ to $\theta _{H}$. The relationship between them can be given by minimization of the ferromagnetic free energy. The free energy *F* is the sum of three terms^23^,

18 $$\begin{aligned} F=-\vec {H} \cdot \vec {M}+H_{an} M_{s} \sin \frac{\xi _{a}}{2}+H_{d} M_{s} \cos ^{2} \theta , \end{aligned}$$

where $\vec {M}=M_{s} \vec {m}$, $M_{s}$ is saturation magnetization, and $\vec {m}$ is a unit vector, indicating the direction of magnetization. $H_{an}$ is the magnetocrystalline anisotropy field, which is confined in the plane of the layer. $H_{d}$ is the demagnetizing field. And $\xi _{a}=\left( \vec {H}_{an},\vec {m}\right)$ is the angle between the magnetocrystalline anisotropy axis and the magnetization. The relationship between the magnetization direction $\theta$ and the direction of the external magnetic field $\theta _{H}$ can be obtained by solving the minimum free energy. And as a result, the relationship between $\theta$ and $\theta _{H}$ is shown in Fig. 3. It is seen that $\theta$ varies around $\theta =\theta _{H}$. Once we have the relationship between $\theta$ and $\theta _{H}$, we can bring it into Eq. (17) and fit our theoretical curve with the experimental data. In Fig. 4, we give the fitting curves of Pt and Cu respectively in the out-of-plane configuration. It is seen that our formula fits the data very well.

### Magnetization in x–z plane

When magnetization in x–z plane, we have $\varphi _{H}=\varphi$ = 0 °C. This case was not tested in experiments^23^. However it is instructive to exhibit the variation of the output voltage drop on the angle of magnetization in x–z plane. The voltage in the *y* direction is then

19 $$\begin{aligned} \Delta V_{y} & = A \Delta S\left\{ \left[ -\left( (pt)^{s} r-\Pi ^{s} r_{RL}\right) \frac{J_{x}^{s}}{\left( h/2\pi \right) }-r_{RL} J_{x}^{q}\right] \cos \theta \right. \nonumber \\&\quad +\left. \left[ \left( \Pi ^{s} r+(pt)^{s} r_{RL}\right) \frac{J_{y}^{s}}{\left( h/2\pi \right) }\right] \right\} . \end{aligned}$$

The first term in the factor of $\cos \theta$ term, i.e. $-\left( (pt)^{s} r-\Pi ^{s} r_{RL}\right) \frac{J_{x}^{s}}{\left( h/2\pi \right) }$, is related to spin caloritronic effects completely. While the second term, $r_{RL} J_{x}^{q}$, is related to heat flowing without spin effects. The last term of Eq. (19) is also dependent on spin effects. Therefore, the formula returns to a $\cos \theta$ behavior in which voltage should be zero at $\theta =90^{\circ }$. The existence of spin effects will shift voltage from zero. This can be a test of existence of spin effect in further experiments. To express the argument more clearly, we factorize the formula by $\Delta V_{y}=V_{1}\cos \theta +V_{0}$, and show the variation of $\Delta V_{y}$ on $\theta$ in Fig. 5. With increasing the parameter $V_{0}/V_{1}$, the voltage output deviates from zero at $\theta$ = 90 °C more apparently. We hope this can be tested in future experiments.

## Discussion and conclusion

Wegrowe et al. found an angular dependence between the transverse temperature gradient and the direction of magnetization (formula (16) in Ref.^25^) based on the magnons transport. In this paper, we also obtain an angular dependence between them in ferromagnetic metals based on electron transport (Eq. (15)). Comparing Eq. (15) to Eq. (16) in Ref.^25^, there are two extra terms of $(\sin \theta \sin \varphi )^{2}$-angle term and a term independent of magnetization angles. These terms stem from extra spin caloritronic effects which can not be captured in a formalism based on magnon transport. Since $\hbox {Ni}_{{80}}\hbox {Fe}_{{20}}$, a metal, was exploited in experiment^23^, it is more natural to use our formulas derived by electron transport. By fitting the experimental data in Ref.^23^, it is found that introducing spin effects gives rise to a phase shift and our formulas fit the experimental data better. Phase shifts for different materials can be obtained by fitting. This confirms the existence of spin-thermal effects in the anomalous Righi–Leduc effect. Satya et al. found a large net Berry curvature near the Fermi energy of a ferromagnetic metal $\hbox {Co}_2$MnGa. This means that there is likely to be a much larger SNE in this material than in ordinary ferromagnetic metals^15^. Spin effects may be more prominent in this material and the effects predicted in this work may be more easily tested. Our formulas are general and the physical quantities serve as fitting parameters. Therefore they are suitable not only for diffusion regime but also for ballistic regime. In the latter case, spin coherence may be reserved so that the predicted spin effects may be more prominent. Therefore, another way to identify the predicted spin effects is to measure the similar devices which are within the spin diffusion length and smaller than those in the existing experiment^23^.

## Appendix

### Fitting parameters

In this section, we will give the fitting parameters of the solid line in Fig. 2 and the curve in Fig. 4. In Fig. 2 (x–y configuration), the fitting formula we used is $\triangle V_{y}=A \sin \varphi \cos \varphi +B \sin ^{2} \varphi +C$. The fitting parameters are as follows:

$$\begin{aligned} \begin{array}{l}{ \text{ Pt: } A=-0.05814 , B=0.01998 , C=-\,3.41578 \times 10^{-4}}; \\ { \text{ Bi: } A=-\,0.5935, B=0.01965, C=0.02867}; \\ { \text{ Cu: } A=-\,0.07579, B=0.02867, C=-\,0.0022}. \end{array} \end{aligned}$$

In Fig. 4 (y–z configuration), the fitting formula we used is $\bigtriangleup V_{y}=A \cos \theta +B \sin ^{2} \theta +C$. The fitting parameters are as follows:

$$\begin{aligned} \begin{array}{l}{ \text{ Pt: } A=-\,0.2275, B=0.0076, C=-\,0.0084}; \\ { \text{ Cu: } A=-\,1.12805, B=0.06184, C=0.05885.}\end{array} \end{aligned}$$

### Matrices $\hat{\mathbf {P}}_{m}$ and $\hat{\mathbf {Q}}_{m}$

In the following, we derive explicit expressions of the matrices ${\hat{\mathbf {P}}}_{m}$ and ${\hat{\mathbf {Q}}}_{m}$ mentioned in the maintext. ${\hat{\mathbf {P}}}_{m}$ is the direct product of vector $\mathbf {m}$ and vector $\mathbf {m}^{t}$. Where $\mathbf {m}=(\sin \theta \cos \varphi , \sin \theta \sin \varphi , \cos \theta$) is a row vector, and $\mathbf {m}^{t}$ is the transpose of $\mathbf {m}$, which is a column vector. The direct product of them is ${\hat{\mathbf {P}}}_{m}$,

$$\begin{aligned} {\hat{P}}_{m} =\mathbf {m} \otimes \mathbf {m}^{t} = \left( \begin{array}{ccc}{\sin ^{2} \theta \cos ^{2} \varphi } &{} {\sin ^{2}\theta \sin \varphi \cos \varphi } &{} {\sin \theta \cos \theta \cos \varphi } \\ {\sin ^{2}\theta \sin \varphi \cos \varphi } &{} {\sin ^{2}\theta \sin ^{2}\varphi } &{} {\sin \theta \cos \theta \sin \varphi } \\ {\sin \theta \cos \theta \cos \varphi } &{} {\sin \theta \cos \theta \sin \varphi } &{} {\cos ^{2}\theta }\end{array}\right) . \end{aligned}$$

And ${\hat{\mathbf {Q}}}_{m}$ is the antisymmetric representation of $\mathbf {m}$, which is a matrix, corresponding to the operation ${\hat{\mathbf {Q}}}_{m}$: $\mathbf {X}\mapsto \mathbf {m}\times \mathbf {X}$, i.e.

$$\begin{aligned} {\hat{\mathbf {Q}}}_{m}=\left( \begin{array}{ccc}{0} &{} {\cos \theta } &{} {-\sin \theta \sin \varphi } \\ {-\cos \theta } &{} {0} &{} {\sin \theta \cos \varphi } \\ { \sin \theta \sin \varphi } &{} {-\sin \theta \cos \varphi } &{} {0}\end{array}\right) . \end{aligned}$$

### The formula for voltage drop

Thermocouple is often used experimentally to measure temperature difference. Thermocouple measurement of temperature difference is achieved by converting temperature difference into voltage drop, and this voltage drop can be divided into two parts. The first part is the temperature-difference-induced potential $\Delta V_{y}^{(1)}$ at both ends of the measured object (here is the electrode, such as Pt). And the second part is the temperature-difference-induced potential $\Delta V_{y}^{(2)}$ on the wires connected to the voltmeter. Firstly, we calculate the temperature difference potential $\Delta V_{y}^{(1)}$ on the electrode Pt. The linear response in the electrode Pt is $\mathbf {J}=-\frac{\varvec{\sigma }}{q} \varvec{\nabla } \mu ^{e}-\varvec{\sigma } \mathbf {S} \varvec{\nabla } T$. In open circuit situation, we have $\mathbf {J}=0$, thus $0=-\frac{\varvec{\sigma }}{q} \varvec{\nabla } \mu ^{e}-\varvec{\sigma } \mathbf {S} \varvec{\nabla } T$. The *y* component is $-\frac{1}{q}\nabla _{y} \mu ^{e} =S_{N}\nabla _{x} T+S\nabla _{y} T$. Where *S* represents the Seebeck coefficient of Pt electrode and $S_{N}$ represents the Nernst coefficient of the electrode. The integration of electrochemical potential gradients gives us the voltage drop in Pt, i.e. $\Delta V_{y}=-\int _{C}^{D} \frac{\partial \mu ^{e}}{q \partial y} d y$. Where C and D are the two contact points of the electrode and the wires, and both C and D are on the electrode. So the temperature difference potential $\Delta V_{y}^{(1)}$ on the electrode can be written as:

20 $$\begin{aligned} \Delta V_{y}^{(1)}=\int _{C}^{D}S_{N} \frac{\partial T}{\partial x}dy+\int _{C}^{D}S \frac{\partial T}{\partial y}dy. \end{aligned}$$

We assume that *S* and $S_{N}$ are independent of temperature *T*, so the above formula can be written as:

21 $$\begin{aligned} \Delta V_{y}^{(1)}=S_{N}\Delta _{x} T + S \Delta _{y}T, \end{aligned}$$

where $\Delta _{x} T=\int _{C}^{D} \frac{\partial T}{\partial x} d y$, $\Delta _{y} T=\int _{C}^{D} \frac{\partial T}{\partial y} d y$. Similarly, we can get the expression of the temperature difference potential $\Delta V_{y}^{(2)}$ on the wires. But in the derivation, since we consider that the wires are very thin and narrow (a wire), we ignore the transverse effect on the wires. So, the temperature difference potential $\Delta V_{y}^{(2)}$ on the wires is

22 $$\begin{aligned} \Delta V_{y}^{(2)}=\int _{C}^{D}S^{\prime } \frac{\partial T}{\partial y}dy= S^{\prime } \Delta _{y}T, \end{aligned}$$

where $S^{\prime }$ is the Seebeck coefficient of the wires, and, at this point, C and D are still the two points of contact between the electrode and the wires, but now these two points are on the wires. And in the calculation of the above formula, we still assume that $S^{\prime }$ is independent of temperature *T*. Since the temperature-difference-induced potential between the electrode and the wires cancels each other out in the entire circuit, we have

23 $$\begin{aligned} \Delta V_{y}=\Delta V_{y}^{(1)}-\Delta V_{y}^{(2)}=\Delta S \Delta _{y} T+S_{N} \Delta _{x} T, \end{aligned}$$

where $\Delta S$ is the Seebeck coefficient difference between the electrode and the wires , i.e. $\triangle S =S-S^{\prime }$.

### Table for parameters

For convenience, we made a table of the different coefficients and the names of the effects they are usually associated with. As shown in Table 1.

**Table 1:** Various effects and associated physical quantities are tabulated.

$\kappa$	Thermal conductivity (x, y direction)
$\kappa _{z}$	Thermal conductivity (z direction)
$\lambda _{RL}$	The anomalous Righi–Leduc coefficient
$\Pi ^{s}$	The spin Peltier coefficient (x, y direction)
$\Pi _{z}^{s}$	The spin Peltier coefficient (z direction)
$(pt)^{s}$	A transverse effect coefficient, corresponding to the transverse heat flow caused by the longitudinal spin flow
$r=\kappa /[\kappa ^{2}+(\lambda _{RL})^{2}]$	Thermal resistivity (x, y direction)
$r_{z}=1/\kappa _{z}$	Thermal resistivity (z direction)
$\Delta r =(1/\kappa _{z})-r$	The anisotropy of the thermal resistance (z direction)
$r_{RL}=\lambda _{RL}/[\kappa ^{2}+(\lambda _{RL})^{2}]$	Righi–Leduc resistivity (z direction)
